# Retrospective analysis of treatment outcome in 315 patients with oligodendroglial brain tumors

**DOI:** 10.1186/1471-2377-9-33

**Published:** 2009-07-16

**Authors:** J Vesper, E Graf, C Wille, J Tilgner, M Trippel, G Nikkhah, CB Ostertag

**Affiliations:** 1 Department of Functional Neurosurgery, Neurosurgical Clinic, Heinrich-Heine University Duesseldorf, Germany; 2 Department of Biometry and Data Management, Center for Clinical Studies, University of Freiburg, Germany; 3 Department of Stereotactic Neurosurgery, Neurocenter, University of Freiburg, Germany

## Abstract

Although chemotherapy with procarbazine, lomustine and vincristine (PCV) is considered to be well tolerated, side effects frequently lead to dose reduction or even discontinuation of treatment of oligodendroglial brain tumors. The primary objective of the analysis was to retrospectively compare progression-free survival (PFS) after PCV vs. PC chemotherapy (without vincristine to avoid side effects). Patients were retrospectively identified from a database containing our patients between 1990 and 2003. For the selected cases, all histopathology reports were re-evaluated by a local neuropathologist. Based on the updated histology data, patients were included in the study if they had at least one histological diagnosis of an oligodendroglial tumor. PFS after start of PCV (n = 61) and PC (n = 84) chemotherapy identical (median 30 months). Multivariate analysis adjusting for prognostic imbalances favouring the PC group showed a minor, statistically non-significant benefit for PCV (hazard ratio 0.81, 95% confidence interval 0.53–1.25; p = 0.346). Younger age (< 50 y) was a statistically significant predictor of longer PFS. Significant advantages in terms of overall survival after first diagnosis of oligodendroglial tumor (OS, n = 315) were found for patients < 50 y (p < 0.001), oligodendrogliomas versus oligoastrocytomas (p = 0.002), and WHO°II vs. °III (p < 0.001). Three risk groups regarding OS were identified. Findings support the hypothesis that PC may be as effective as PCV chemotherapy, while avoiding the additonal risks of vincristine. Younger age, lower tumor grade and histology of an oligodendroglioma were identified to be favorable prognostic factors.

## Background

Oligodendroglial brain tumors constitute a small subgroup of tumors of the central nervous system. They belong to the group of glial tumors. Histologically, so-called pure oligodendrogliomas are usually composed of a single cell type. Mixed gliomas, so-called oligoastrocytomas, additionally contain astrocytes. The WHO classification [1] distinguishes lowly malignant grade II tumors and anaplastic grade III tumors. Malignant transformation in the course of disease is common for these tumors, which is why affected patients have a reduced life expectancy. Median survival of patients with astrocytic brain tumors has been reported to be about 3.9 years for WHO grade II tumors and about 2.8 years for WHO III tumors [2-11]. Since PCV chemotherapy (procarbazine, CCNU, vincristine) became available as a new therapeutic option in addition to radiotherapy and surgical resection, neuro-oncologists have shown an increasing interest in oligodendroglial tumors in recent years.

Published studies on oligodendrogliomas are very heterogeneous in terms of patient numbers, treatment modalities and histological features and therefore difficult to compare. Many investigators have confirmed the prognostic value of 1p/19q loss and have shown that this change occurs very early in the course of tumor development [12-16]. The efficacy of available therapies is still limited despite new insights into the molecular structure of oligodendrogliomas. Temozolomide is typically the first-line drug for oral chemotherapy because it is assumed to have fewer side effects than the PCV regimen. The first clinical study directly comparing temozolomide and PCV treatment in patients with malignant oligodendroglial tumors is currently underway [17-19]. However, the standard combination of procarbazine, CCNU and vincristine is associated with a high number of severe side effects. The main side effects of vincristine are peripheral neuropathy and constipation. Vincristine binds to tubulin dimers causing disassembly of microtubules. Disruption of the microtubules arrests mitosis in metaphase. The vinca alkaloids therefore affect all rapidly dividing cells, including cancer cells but also the intestinal epithelium and bone marrow. Based on the clinical impression that vincristine was responsible for many side effects and had no additional benefit for the patients, treatment in Freiburg was changed to therapy without vincristine (PC chemotherapy).

The primary objective of this study was to retrospectively compare progression-free survival (PFS) after PCV chemotherapy versus PC chemotherapy according to the Freiburg regimen, which was introduced in 1996. Adjustments were made for age, previous resection, histological type and grade, and Karnofsky performance score (KPS). PFS after resection was evaluated descriptively. In addition, overall survival from first diagnosis of oligodendroglioma or oligoastrocytoma was explored in relation to prognostic factors.

## Methods

### Patients

Patients were retrospectively identified from the MS ACCESS database of our department containing all patients treated since Jan. 1, 1990. The cut-off for inclusion in the study was Dec. 31, 2003. Patients were selected for review if they had at least one diagnosis of oligodendroglioma or oligoastrocytoma. Patients gave their informed consent for data acquisition. The study was approved by the local ethical committee. For the selected cases, all histopathology reports and specimens removed at our department were re-evaluated by a local neuropathologist according to current standards. Based on the updated histology reports, patients were included in the study if they had at least one histological diagnosis of an oligodendroglial tumor, i.e. WHO grade II or III oligodendroglioma or WHO grade II or III oligoastrocytoma, and if follow-up data were available. Of 369 patients who fulfilled the initial criteria, 54 were excluded either because histological re-evaluation failed to confirm oligodendroglial tumor or because no follow-up was available, leaving 315 patients for inclusion in the analysis.

### Staging and grading

Patients underwent stereotactic serial biopsies as previously described [20]. Intraoperatively, specimens were obtained for smear preparation and/or paraffin conservation. A mixed oligodendroglioma was diagnosed when the tumor contained at least 25% of oligodendrocytic elements. For the purpose of this study, grading and staging of the tumors were postoperatively re-evaluated by a board-certified neuropathologist [20]. If a follow-up biopsy rated a tumor as more benign than previous biopsies, the more recent benign diagnosis was retrospectively assumed for the earlier biopsies as well. This occurred in 5 patients and 5 biopsies.

### Treatments

Patients with anaplastic oligoastrocytomas or oligodendrogliomas (WHO grade III) usually underwent radiotherapy with a maximum dose of 60 Gy in 30 daily fractions within the first 6 weeks after surgery. The planned target volume was variable and was defined on the basis of postoperative magnetic resonance imaging (MRI) or computed tomography (CT) findings.

PCV chemotherapy consisted in standard cycles of 110 mg/m^2 ^of oral lomustine on day one (maximum of 200 mg) together with ondansetron (domperidone or metocopramide) as an antiemetic drug, followed by 60 mg/m^2 ^of oral procarbazine on days 8 through 21, and 1.4 mg/m^2 ^of IV vincristine on days 8 and 29 (maximum of 2 mg/day). Cycles were repeated every 6 weeks. PC chemotherapy was used in single cases starting in 1996 and has been in routine use since 2000. The dosages of lomustine and procarbazine were the same, the cycles were extended to 12 weeks.

PCV and PC chemotherapy were continued for at least 3 cycles. Tumor response was evaluated by means of contrast-enhanced imaging (CT or MRI) every three months and therapy discontinued if there was tumor regression or stable disease according to MacDonald criteria for at least 6 months [21]. In addition chemotherapy was readjusted or discontinued in case of hematological or neurological toxicity.

Seed implantation was used instead of fractionated radiotherapy to treat tumors with a maximum diameter of 30 mm and a spherical configuration. I^125 ^seeds in a 5-mm titanium capsule were temporarily implanted for 20–30 days to reach the 60 Gy isodose at the tumor margin.

Microsurgical resection was typically performed on tumors not involving the basal ganglia or eloquent regions of the brain, and only exceptionally for recurrent tumors not responding to chemotherapy and radiation therapy.

### Follow-up and evaluation criteria

After the end of treatment, follow-up examinations including contrast-enhanced MRI or CT were performed at 6-month intervals. For patients not followed up at our hospital, follow-up dates, times of progression, therapies in other hospitals and survival data were sought using both a questionnaire, which was sent to the patients, and telephone calls to the caretakers. Information on the last follow-up of surviving patients was obtained in the period between June 2004 and December 2005.

Progression-free survival was the primary endpoint, defined as the interval from initiation of treatment (PCV, PC, resection or seed implantation) to the next progression or death. Based on the available data, progression was retrospectively determined as either image-defined progression (>25% contrast-enhanced tumor) or initiation of subsequent treatment (chemotherapy, radiation, resection or seed implantation ≥ 4 weeks after start of treatment; treatments within 4 weeks were interpreted as planned interventions). For comparison of PCV (n = 61) versus PC (n = 84), patients were retrospectively assigned to the treatment groups based on the regimen they received first, excluding 167 patients who received neither PCV nor PC after diagnosis of oligodendroglioma or oligoastrocytoma, and 3 patients whose follow-up ended with the administration of chemotherapy. PFS of patients with resection (n = 98) was evaluated descriptively. Overall survival (OS) was defined as time from first diagnosis of oligodendroglioma or oligoastrocytoma to death from any cause and was assessed in the entire group of patients.

### Statistical methods

PFS and OS were estimated by the Kaplan-Meier method, censoring observations at the time of last follow-up if the respective event was not observed [22]. Median follow-up was determined using the reverse Kaplan-Meier estimator [23]. Prognostic factors (age </≥50 years) [24], previous resection, histological type and grade, Karnofsky performance status (</≥90) [25], and a treatment indicator (where applicable) were all fitted together in pre-specified Cox regression models. In addition, the treatment indicator in the PCV versus PC comparison was subjected to exploratory sensitivity analyses to study the potential effect of correlation between treatment and other factors. For the OS model a prognostic score was calculated for each patient. Kaplan-Meier plots were produced for three risk groups of equal size with low, medium and high risk. Toxicity rates were compared by Fisher's exact test. Two-sided 95% confidence intervals were used and statistical significance defined as p < 0.05 based on two-sided tests.

## Results

After re-evaluation of histological findings, 315 patients with oligodendroglial tumors and follow-up data were available for analysis. The majority of patients were younger than 50 when they were first diagnosed with an oligodendroglial tumor. Sixty-four percent of the patients (n = 202) were men. At the time of diagnosis, 26% of patients (n = 83) had already undergone tumor resection. The histological diagnoses were grade II oligodendroglioma in 28% (n = 87), grade III oligodendroglioma in 3% (n = 10), grade II oligoastrocytoma in 57% (n = 181), and grade III oligoastrocytoma in 12% (n = 37). In two patients with oligoastrocytoma definitive grading was not possible (Table 1).

**Table 1:** Description of patient population at the time of first diagnosis of oligodendroglioma or oligoastrocytoma (n = 315)

		n	%
Age (in years)	<20	15	5%
	≥20 – <30	53	17%
	≥30 – <40	87	28%
	≥40 – <50	82	26%
	≥50 – <60	43	14%
	≥60 – <70	26	8%
	≥70	9	3%

	median (range)	40 (4 – 77)	

Sex	male	202	64%
	female	113	36%

Previous resection	yes	83	26%
	no	232	74%

Histology and grade	Oligodendroglioma, WHO II	87	28%
	Oligodendroglioma, WHO III	10	3%
	Oligoastrocytoma, WHO II	181	57%
	Oligoastrocytoma, WHO III	37	12%

In the total study population of 315 patients, 145 patients with a diagnosis of oligodendroglial tumor were treated by PCV (n = 61) or PC (n = 84) chemotherapy. In three additional cases, follow-up ended with the completion of chemotherapy. PCV chemotherapy was used from 1994 to 2001; PC chemotherapy was used only in individual patients before 2000 and then routinely until the end of the observation period in 2005. 9 patients underwent PC chemotherapy after PCV was discontinued due to toxicity. They were included in the PCV group. Age and sex distribution were comparable in both treatment groups (Table 2). Oligodendroglioma was present in about a quarter of both PCV and PC patients, respectively. Median time from diagnosis of oligodendroglioma to onset of chemotherapy was 2.8 (PVC) versus 0.4 years. Histological grading and Karnofsky performance status (KPS) at initiation of chemotherapy were better in PC patients, with WHO grade II in 52% (PCV, n = 32) versus 67% (PC, n = 56), and KPS 90 or 100 in 59% (PCV, n = 35) versus 66% (PC, n = 55). 19% (n = 31) of PCV and 23% (n = 19) of PC patients had received radiotherapy or a seed implantation before PC(V). Tumor resection had been performed before chemotherapy in 49% (n = 30) of patients in the PCV group versus 27% (n = 23) in the PC group. Surgical resection was mostly performed in patients with larger tumors and significant mass effect prior to surgery but its effect is controversial [26]. Therefore it is not clear a priori which group is favored by the imbalance with respect to resection.

**Table 2:** Description of patient population at time of PCV or PC treatment

		PCV n = 61		PC n = 84	
Age (in years)	< 50	40	(66%)	53	(63%)
	≥50	21	(34%)	31	(37%)

	median (range)	44 (14 – 73)		42 (19 – 77)	

Sex	male	38	(62%)	54	(64%)
	female	23	(38%)	30	(36%)

Time from diagnosis of oligodendroglioma to onset of chemotherapy (in years)	median (range)	2.8 (0 – 17)		0.4 (0 – 18)	

Resection prior to chemo-therapy	yes	30	(49%)	23	(27%)
	no	31	(51%)	61	(73%)

Radiotherapy or seed implantation prior to chemotherapy	yes	30	(49%)	54	(64%)
	no	31	(51%)	65	(36%)

Histology and grade	Oligodendroglioma, WHO II	10	(16%)	16	(19%)
	Oligodendroglioma, WHO III	6	(10%)	7	(8%)
	Oligoastrocytoma, WHO II	22	(36%)	40	(48%)
	Oligoastrocytoma, WHO III	21	(34%)	18	(21%)
	Other, WHO III/IV°	2	(3%)	3	(4%)

Karnofsky performance score	< 90	24	(41%)	28	(34%)
	≥90	35	(59%)	55	(66%)
	Unknown	2		1	

**Figure 1:**
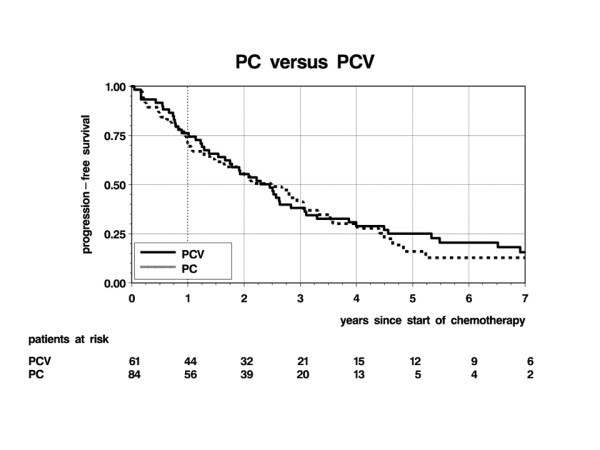
**Progression-free survival for PCV (n = 61) vs PC (n = 84)**.

Regarding outcome, 102 events (85 progressions, 17 deaths) were observed with respect to PFS after a median follow-up time of 6 years. The crude PFS rates of PCV and PC patients were nearly identical over the first four years following start of treatment (Figure 1). Median PFS was 2 years 6 months after both PCV and PC (95% confidence interval [95%-CI]: 1 year 8 months – 3 years 1 month). The adjusted hazard ratio, which corrects for the above-mentioned imbalances, was 0.81 for PCV versus PC (95%-CI: 0.53–1.25; p = 0.35), favoring PCV. Young age, no previous resection, oligodendroglioma, WHO grade II and KPS ≥ 90 were factors associated with good prognosis, with previous resection reaching statistical significance (p = 0.047) and age borderline significance (p = 0.051; Table 3). In an additional exploratory investigation of the stability of the estimated hazard ratio for PCV versus PC, sensitivity analyses studying effects in subgroups defined by WHO grade, KPS and resection were performed. The resulting PCV versus PC hazard ratios within subgroups (adjusted for the other factors) ranged between 0.62 for WHO grade III tumors and 1.04 for patients without previous resection, but none was statistically significant (data not shown).

**Table 3:** Determinants of progression-free survival after PCV (n = 59) and PC (n = 83) treatment in patients with complete data (100 events)

		Hazard ratio*	95% confidence interval	p value
Treatment	PCV vs. PC	0.81	0.53–1.25	0.346

Age (in years)	< 50 vs. ≥50	0.64	0.41–1.00	0.051

Resection before chemotherapy	yes vs. no	1.61	1.01–2.58	0.047

Histology	Oligoastrocytoma vs oligodendroglioma	1.34	0.83–2.18	0.487
	Other vs oligodendroglioma	1.42	0.46–4.39	

WHO grade	II vs III/IV	0.75	0.49–1.14	0.176

Karnofsky performance score	< 90 vs ≥90	1.26	0.81–1.96	0.304

* A hazard ratio < 1 (> 1) indicates an effect in favor of the first (the second) group in terms of progression-free survival.

**Figure 2:**
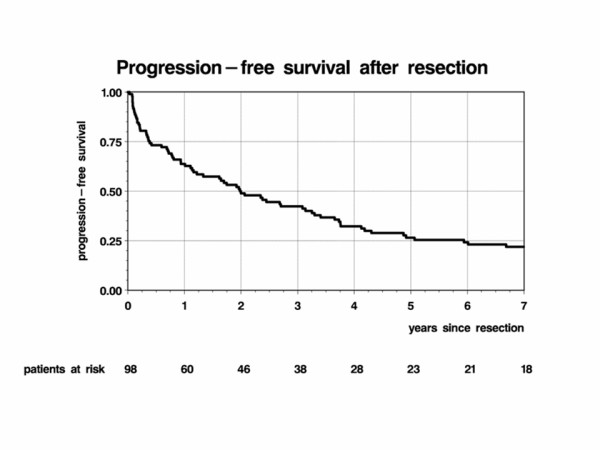
**Progression-free survival after resection (n = 98)**.

PFS after chemotherapy was significantly shorter in patients with prior resection (hazard ratio 1.61, 95%-CI: 1.01–2.58, p = 0.047; Table 3). Figure 2 shows the PFS rates after resection in 98 patients. Median time to progression or death was 2 years (95%-CI: 1 year 2 months – 3 years 2 months; 81 progressions, 10 deaths).

Evaluation of toxicity revealed a predominance of neurological symptoms in the PCV group. There was no difference in the occurrence of Grade 2, 3 and 4 side effects in further parameters except white blood cell count (Table 5).

**Table 5:** NCI Common Toxicity Criteria for PCV and PC treated patients (n = 84 vs. n = 61 respectively)

**Category**	**Regime**	**Toxicity**	**Grade 2**		**Grade 3**		**Grade 4**		**p-value**
**Neurological**	*PC*	*Sensory*	*0*	*0%*	*0*	*0%*	*0*	*0%*	*0.002*
				
	*PCV*		*10*	*12%*	*3*	*(4%)*	*0*	*(0%)*	
	
	PC	Motor	0	(0%)	0	(0%)	0	(0%)	0.26
				
	PCV		3	(4%)	0	(0%)	0	(0%)	

**Gastrointestinal**	*PC*	*Nausea*	*15*	*(25%)*	*3*	*(5%)*	*0*	*(0%)*	*0.65*
				
	*PCV*		*15*	*(18%)*	*5*	*(6%)*	*0*	*(0%)*	
	
	PC	Vomiting	10	(16%)	4	(7%)	0	(0%)	0.62
				
	PCV		9	(11%)	5	(6%)	0	(0%)	

**Blood/Bone Marrow**	*PC*	*WBC*	*35*	*(57%)*	*15*	*(25%)*	*1*	*(2%)*	* < 0.001*
				
	*PCV*		*25*	*(30%)*	*14*	*(17%)*	*2*	*(2%)*	
	
	PC	PLT	10	(16%)	8	(13%)	2	(3%)	0.20
				
	PCV		8	(10%)	6	(7%)	1	(1%)	
	
	*PC*	*Hgb*	*7*	*(11%)*	*5*	*(8%)*	*1*	*(2%)*	*0.24*
				
	*PCV*		*6*	*(7%)*	*3*	*(4%)*	*0*	*(0%)*	

**Allergy**	PC	Allergy	6	(10%)	0	(0%)	0	(0%)	0.33
				
	PCV		4	(5%)	2	(2%)	0	(0%)	

**Figure 3:**
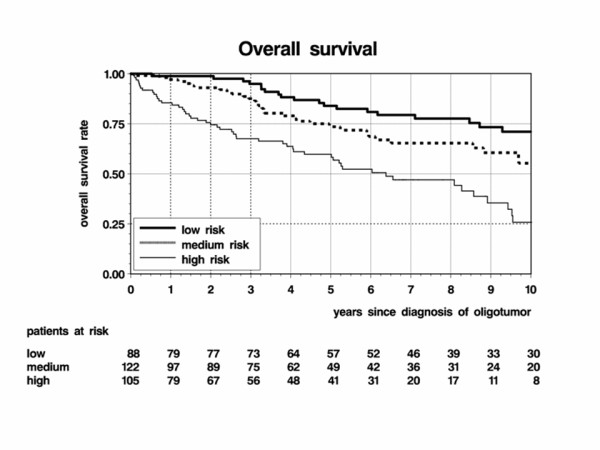
**Overall survival after first diagnosis of oligodendroglioma or oligoastrocytoma (n = 315) by risk group (low, medium, high risk)**.

In the entire population of 315 patients, 120 deaths occurred during a median follow-up period of 7 years for OS after first diagnosis of oligodendroglial tumor. 88%, 77%, 67%, 63% and 52% of patients survived for more than 2, 4, 6, 8, and 10 years, respectively. Young age, oligodendroglioma and WHO grade II showed large and statistically significant favorable effects on OS, while no relevant influence could be demonstrated for resection (Table 4). Three groups of patients were identified as low, medium and high risk groups. Patients aged under 50 with oligoastrocytoma of WHO grade II and no resection constituted the largest group (n = 122) with medium risk. Those either aged ≥ 50 or aged under 50 with WHO grade III tumors were at high risk (n = 105). Patients were at low risk if they were aged under 50, had a WHO grade II tumor and either a previous resection or oligoastrocytoma but no previous resection (n = 88, Figure 3).

**Table 4:** Determinants of overall survival after first diagnosis of oligodendroglioma or oligoastrocytoma (n = 315 patients, 120 deaths)

		Hazard ratio*	95% confidence interval	p-value
Age (in years)	< 50 vs ≥50	0.40	0.27–0.59	< 0.001

Previous resection	yes vs no	0.87	0.57–1.32	0.506

Histology	Oligoastrocytoma vs Oligodendroglioma	1.99	1.28–3.10	0.002

WHO grade	II vs III	0.39	0.24–0.61	< 0.001

* A hazard ratio < 1 (> 1) indicates an effect in favor of the first (the second) group in terms of overall survival.

## Discussion

The significance of the oligodendroglial tumor component in the histopathological diagnosis has been increasingly recognized with the advent of new chemotherapeutical options over the last decade. In general, patients with oligodendroglial tumors have better therapeutic options and a better prognosis compared to patients with purely astrocytic tumors. However, it is assumed that the more favorable prognosis of these patients is crucially determined by the treatment performed. To investigate the effects of different therapies on prognosis, we undertook this retrospective analysis of 315 consecutive patients diagnosed with an oligodendroglial tumor at our department from 1990 to 2003. Our study population is comparable to the patient populations investigated in other large studies in terms of age distribution and frequency of histological diagnoses [27,28].

Another aim of the retrospective analysis was to find out whether combined chemotherapy with procarbazine and CCNU without vincristine may be equally effective even when the cycle of administration is prolonged. The crude progression-free survival rates found in our patients after initiation of PCV and PC therapy are indeed very similar, while more detailed analyses adjusting for some prognostic imbalances which favored the PC group show a minor and statistically non-significant treatment benefit for PCV. Obviously, these results need to be interpreted with care. In addition to the absence of randomization, the time-lag of nearly a decade between PCV and PC treatment of our patients introduces a great potential to bias treatment comparisons. During this period a lot more than chemotherapy treatment has been changed. However, the study does add some evidence to the hypothesis that vincristine administration may be unnecessary. Another advantage of the PC regimen is that it allows completely oral administration and can therefore also be performed on an outpatient basis.

PFS after PCV versus PC was determined from the start of chemotherapy, independent of previous treatments. Tumor progression was assumed if a new therapy was initiated later than four weeks after preceding treatment assuming retrospectively that this was done for recurrent tumor while a second therapy within the first four weeks was interpreted as a planned intervention. However, since we analyzed the data retrospectively, standardized and reliable radiological follow-up data are not available in all cases. Therefore, our results need to be validated further in prospective studies. The median interval until progression after tumor resection was only two years (Figure 2). Overall survival of patients who already underwent resection before the first diagnosis of an oligodendroglial tumor did not differ from that of the remaining patients (Table 4). Taking in account latest published data on the prognostic importance of total resection, we have to consider a strong bias within the resection group. The extent of resection was not evaluated. Patients underwent surgery mostly in cases with severe mass effects. Therefore results (Table 3 and Figure 2) must not allow one to conclude anything about the value of resection but rather suggest that the overall prognosis of these tumors is influenced by numerous additional factors. Furthermore, genetic factors, such as 1p19q constellations have been identified as favorable prognostic factors for temozolomide treatment of oligodendroglial tumors [29]. More evaluations are required to elucidate this question.

A lower tumor grade (WHO grade II) and younger age were significantly associated with a favorable prognosis regarding OS after first diagnosis of oligodendroglial tumor. This observation is in agreement with earlier reports [30,31].

The disadvantages of Vincristine might be avoidable. PCV leads to significantly more neurological symptoms than PC alone (Table 5). This corresponds to the data for PCV administration of other authors [32].

## Conclusion

In summary, our retrospective results show the benefits of combined therapeutic approaches in patients with oligodendroglial tumors. When carefully considering an individual patient's situation, combined chemotherapy with PC appears to be just as effective as conventional PCV therapy. In recent years, temozolomide has been increasingly used because of its lower rate of side effects. However, results of a direct comparison of temozolimide with established chemotherapeutic regimens are still lacking.

## Pre-publication history

The pre-publication history for this paper can be accessed here:


